# Impact of Fetal and Infant Exposure to the Chinese Great Famine on the Risk of Hypertension in Adulthood

**DOI:** 10.1371/journal.pone.0049720

**Published:** 2012-11-21

**Authors:** Pei-Xi Wang, Jia-Ji Wang, Yi-Xiong Lei, Lin Xiao, Zhong-Cheng Luo

**Affiliations:** 1 Department of Preventive Medicine, School of Public Health, Guangzhou Medical University, Guangzhou, China; 2 Department of Obstetrics and Gynecology, CHU Sainte-Justine, University of Montreal, Montreal, Canada; Fudan University, China

## Abstract

**Background:**

Famine provides quasi-experimental conditions for testing the hypothesis of “programming” health effects by poor nutrition in early life. It remains uncertain whether early life exposure to famine increases the risk of hypertension in adulthood. There is a lack of data on the relative impact of exposure to famine during fetal development versus infancy (<2 years postnatal). We sought to assess the impact of exposure to the 1959–1961 Chinese Great Famine (the largest in human history) during fetal development and infancy on the risks of hypertension, short stature and obesity in adulthood.

**Methodology/Principal Findings:**

We conducted a retrospective cohort study of 12,065 adults (46–53 years of age) born 1957–1964 in the Zhongshan and Nanhai municipalities of Guangdong province, China. Adjusting for socio-demographic and lifestyle characteristics, as compared to subjects who were unexposed to famine, the risk of hypertension was not significantly elevated in subjects exposed to famine during fetal development only overall, but was 1.36-fold higher in those exposed during the first trimester of pregnancy only [adjusted odds ratio (OR) 1.36 (95% confidence intervals 1.03–1.79)], 1.83-fold higher in those exposed during infancy only [adjusted OR 1.83 (1.61–2.08)], and 1.31-fold higher in those exposed during both fetal development and infancy [adjusted OR 1.31 (1.14–1.51)]. Exposure to famine during infancy increased the risk of short stature. Early life exposure to famine did not increase the risk of obesity.

**Conclusions/Significance:**

Exposure to the Chinese Great Famine during the first trimester of pregnancy only, or during infancy only, or during both fetal development and infancy increased the risk of hypertension in adulthood, suggesting an important role of changes in exposure to famine during fetal development and from prenatal to early postnatal life in developmental “programming” cardiovascular disease risk.

## Introduction

Susceptibility to certain degenerative chronic diseases such as hypertension and type 2 diabetes may be “programmed” by poor nutrition in early life [1], [2]. Poor fetal and/or early postnatal nutrition during critical growth phases may alter the structural and physiologic functional development of vital organs regulating blood sugar and blood pressure, thus “programming” the susceptibility to hypertension and diabetes in adulthood [2], [3]. Animal studies have provided strong evidence that poor fetal nutrition leads to low birth weight in offspring and increased blood pressure in adulthood [4], [5]. However, human studies have been largely limited to retrospective cohort studies which could not determine the cause of low birth weight – whether it was due to poor fetal nutrition, a suspected programming driver, or other causes. Poor fetal nutrition may be the key programming driver through establishing a “thrifty” phenotype that selectively protects the growth of the most vital organs (ex. brain) at the expense of the less vital ones (ex. pancreatic β-cell mass, nephron cell mass) during fetal development [2]. Such fetal adaptations increase the chance of survival in an adverse intrauterine environment, but impair metabolic functional capacity in coping with metabolic stressors in postnatal life [2]. Another hypothesis is that a “mismatched” rich nutrition in early postnatal life following poor fetal nutrition may be the detrimental programming driver [6].

Famine provides quasi-experiment conditions to examine the direct evidence for the “programming” of health effects by poor fetal or early postnatal nutrition in humans. Most studies on the health effects of early life exposure to famine have been carried out among subjects exposed to the Dutch Famine in World War II [7]–[12]. The findings are inconsistent concerning whether fetal exposure to the Dutch famine was associated with hypertension or increased blood pressure; some studies reported a positive association [11], but others did not [7], [10]. A study of 1339 adults born before, during or after the civil war (1967–1970) in Nigeria reported significantly increased risk of hypertension in subjects exposed to the war-inflicted famine during fetal and early postnatal life [13]. In contrast, in a study of 169 adults who were exposed in *utero* to malnutrition due to the siege of Leningrad in World War II, no statistically significant difference in blood pressure was observed as compared to 188 subjects who were born outside the area under siege [14]. We speculated that these inconsistent findings could be due to differences in population-specific long-term nutritional history that may have influenced the responses to nutrient deprivation in the womb or in the infancy, or that infants exposed to famine during fetal life might have been “de-programmed” in early postnatal life due to better infant nutrition because the Dutch famine was relative short (November 1944– April 1945) and occurred in an otherwise well-nourished population. Indeed, early life “de-programming” of elevated blood pressure following fetal programming has been observed in animal models [15]. Due to the relatively short duration of the Dutch famine, it is difficult to distinguish the joint impact of exposure to famine during fetal development with versus without subsequent exposure to famine during infancy on the risk of hypertension in adulthood.

The *1959–1961* Chinese Great Famine, caused by a sharp drop in crop production and the “great leap forward” policies, was the largest in human history, lasting approximately 3 years and resulting in about 30 million deaths and about the same number of lost or postponed births [16]. In contrast to the relatively brief Dutch famine, the exceptionally long Chinese famine provides a unique opportunity to examine the “programming” effects of poor nutrition during fetal development versus infancy. It was only until recent years that researchers began to explore the long-term health impact of early life exposure to the Chinese Great Famine. There have been two studies on early life exposure to the Chinese Great Famine in relation to hypertension in adulthood: one study found an association with exposure to famine during fetal development [17], but the other did not [18]. However, due to insufficient information for clearly defining the duration of famine in the study areas, neither study distinguished between subjects who were exposed to famine during fetal development only versus those who were exposed during both fetal development and infancy. The *relative* impact of exposure to famine during fetal development versus infancy on the risk of hypertension remains unexplored. In a large cohort of subjects recruited at around 50 years of age, the primary objective of this study was to clarify the relative impact of exposure to the Chinese Great Famine during fetal development versus infancy on the risk of hypertension in adulthood.

## Methods

### Design and Participants

This study was based on a recent large cross-sectional health survey in the Nanhai and Zhongshan municipalities of Guangdong province, China, from March 2010 through September 2010. The survey was a random sample of 5% of the population of local residents in the two municipalities. The sampled population is similar to the total population in demographic and socioeconomic characteristics (age, sex, education, occupation). Data on current health, medical history, socio-demographic characteristics and lifestyle factors were collected from participants by trained research staff using a structured data collection questionnaire in a home visit. To investigate the effects of exposure to the Chinese Great Famine during fetal development and infancy (<2 years postnatal) on the risk of hypertension, subjects born in the study areas (Nanhai and Zhongshan) in 1957–1964 (the comparable period as in previous Chinese famine studies) were considered in the present study (n = 12,247). Excluding 182 subjects (1.5%) with missing or invalid data on blood pressure, 12,065 subjects remained in the final study cohort.

### Ethics Statement

The study was approved by the research ethics committee of Guangzhou Medical University. Informed consent was obtained from all study participants.

### Exposure to Famine during Fetal Development and Infancy

Overall, Guangdong province was considered a “less severely” affected region during the 1959–1961 Chinese Great Famine, but famine-related deaths remained substantial, as indicated by an estimated 58% increase in the total number of deaths per 1000 population during the famine years as compared to the three preceding years (1956–1958) [19]. Births were less subject to under-reporting and could serve as a more reliable alternative indicator of famine severity. The average number of births per year during the 3 years of famine decreased by 35% in Zhongshan and 40% in Nanhai of Guangdong province, respectively, as compared to the average number of births during the 3 years preceding and the 3 years following the famine.

According to the recollections of local residents of the study areas, severe food shortages started approximately in January 1959, and ended in October 1961 after the autumn harvest when food supplies were restored to approximately the pre-famine levels. Based on recalls of typical food intakes by local residents, estimated average daily energy and protein intakes for adults were about 700–800 kcal and 10–12 grams during the famine period respectively, about 60–70% below the levels of about 1900–2000 kcal and 35–40 grams before or after the famine period respectively. Rice was the predominate source of energy throughout the study period. Due to the lack of detailed birth registration data in China during these years, it is impossible to determine the exact exposure of each individual subject. We therefore defined exposure to famine during fetal development (in utero) and infancy (<2 years postnatal) by month of birth, based on a typical full-term birth at 40 weeks (∼9 months) gestational age. We were interested in the effects of the exposure during the first 2 years of postnatal life because many studies have indicated the critical importance of growth and nutrition during this period in the developmental programming of metabolic and cardiovascular risks [20], [21]. Based on date of birth, subjects were classified into four groups by window of famine exposure – during fetal development only, during infancy only, during both fetal development and infancy, or not exposed to famine in either period (the reference group) (**Table 1**). Subjects born from October 1, 1961 through June 30, 1962 in the 9 months following the end of the famine were considered exposed during fetal development only because they were conceived during the famine but delivered after the famine. Subjects exposed during fetal development only were further sub-grouped by trimester of exposure for exploring the potential differential effects of gestational age window-specific exposure.

**Table 1:** Windows of exposure to the 1959–1961 Chinese Great Famine in the 1957–1964 birth cohort, Zhongshan and Nanhai municipalities, Guangdong province, China.

Date of birth (mm/dd/yyyy)	Exposure to the famine (January 1959 - September 1961)	Age in 2010 (years)
01/01/1957-12/31/1958	Infancy (<2 years postnatal) only	51–53
01/01/1959-09/30/1961	Both fetal development and infancy	49–51
10/01/1961-06/30/1962	Fetal development only	48–49
10/01/1961-12/31/1961	1^st^, 2^nd^ and 3^rd^ trimester	49
01/01/1962-03/31/1962	1^st^ and 2^nd^ trimester	48
04/01/1962-06/30/1962	1^st^ trimester only	48
07/01/1962-12/31/1964	No exposure (reference group)	46–48

### Measurements and Variables

After at least a 20-minutes rest, blood pressure (BP) was measured on the participant’s right arm in seated position to the nearest mmHg according to standard procedures by trained research staff (medical students), in triplicates at least 5 minutes apart during daytime (morning or afternoon) on the date of study interview; the average BP was taken as a continuous outcome measure. Hypertension was defined as diastolic blood pressure (DBP) ≥90 mmHg or systolic blood pressure (SBP) ≥140 mmHg in at least two measurements and confirmed in a follow-up visit, or a previous diagnosis of hypertension by a clinician, or current use of anti-hypertensive medication.

Height to the nearest centimetre (cm) and weight to the nearest 100 grams (g) were measured in duplicates; average values were taken. Short stature was defined as below the 10^th^ percentile of height for sex within the study cohort. Body mass index (BMI, kg/m^2^) was calculated as weight/height^2^. BMI was classified into 4 categories: underweight<18.5, normal weight 18.5–23.9, overweight 24.0–27.9, obese ≥28.0 kg/m^2^, according to criteria recommended for Chinese adults [22], since lower BMI cut-offs are justifiable for identifying subjects at metabolic and cardiovascular risk in Asian versus Western populations [23].

Co-variables included sex, age (years), education (≥high school graduation, yes/no), occupation (involving physical labor in agriculture or industry, yes/no), smoking (yes/no), alcohol use (at least once a month, yes/no), regular physical activity (at least once a week, yes/no), preference for salty foods (yes/no, according to self-declaration), municipality (Zhongshan, Nanhai), and place of residence (rural, urban), according to data collected in the study questionnaire. Ethnicity was not considered because all study subjects are Han (the overwhelmingly dominant ethnicity group in China). Birth weight and gestational age at delivery were unavailable in the study cohort.

The *primary* outcome was hypertension. Secondary outcomes included DBP, SBP, height, BMI, short stature and obesity. We included short stature and obesity as secondary outcomes because previous studies suggested that early life exposure to famine might affect the risks of short stature and obesity [18], [24]–[26] which are related to the risk of hypertension [27], [28].

### Statistical Analysis

Data are presented as mean±standard deviation (SD) for continuous variables, and n (%) for categorical variables. Generalized linear models were applied to obtain the adjusted least square mean (standard error), and adjusted mean difference with 95% confidence interval (CI) in continuous outcomes (blood pressure, height, BMI) between study groups controlling for participant’s characteristics. Logistic regression was used to obtain the crude and adjusted odds ratios (OR) of dichotomous outcomes (hypertension, obesity, short stature). Data analyses were conducted using the Statistical Analysis System (SAS), Version 9.0 (SAS Institute, North Carolina). Two-tailed p values<0.05 were considered statistically significant. The study had a power of 87% to detect a risk ratio of 1.3 or greater for hypertension comparing any famine exposure versus the non-exposed group.

## Results

The results are presented for the whole cohort because results concerning the impact of famine on all outcomes were similar in rural and urban areas, and for the two municipalities. For blood pressure, results on the impact of famine for the two sexes pooled are presented because there were no significant differences by sex. For stature and BMI, results are presented for males and females separately due to sex differences. Except for sex-specific effects on height and BMI, there were no other significant interactions between variables affecting the primary effect estimates of interest.

There were no significant differences between men and women in average age or the prevalence of hypertension (about 14%) in the study population, although the use of anti-hypertensive medication was more frequent in women (4.5%) than in men (3.4%) (**Table 2**). As expected, men had higher average height and weight, were more likely to be a smoker or alcohol user, and were more likely to have completed high school. Women were more likely than men to have a job involving physical labor, and to prefer salty foods. Average SBP, DBP and BMI were slightly but significantly higher in men versus women, while the prevalence rates of overweight (about 26%) and obesity (about 6%) were similar.

**Table 2:** Characteristics of study participants in the 1957–1964 birth cohort from Zhongshan and Nanhai municipalities, Guangdong province, China.

	All subjects (n = 12065)	Men (n = 5916)	Women (n = 6149)	P*
Age (years)	49.1±2.5	49.1±2.4	49.1±2.5	0.89
Education, ≥high school graduation	2233 (18.6)	1392 (23.6)	841 (13.7)	<0.0001
Occupations involving physical labor	5692 (47.2)	2659 (45.0)	3033 (49.3)	<0.0001
Smoking (yes)	3399 (28.2)	3218 (54.4)	181 (2.9)	<0.0001
Alcohol drink (≥1 drink/month)	780 (6.5)	742 (12.6)	38 (0.6)	<0.0001
Physical activity (≥ 1/week)	4130 (34.2)	1950 (33.0)	2180 (35.5)	0.004
Preference for salty foods	2380 (19.7)	1045 (17.7)	1335 (21.7)	<0.0001
Residence in urban areas	3977 (33.0)	1788 (30.2)	2189 (35.6)	<0.0001
Weight (kg)	60.6±9.6	64.9±8.8	56.5±8.3	<0.0001
Height (cm)	161.9±7.5	167.3±5.5	156.9±5.3	<0.0001
Short stature (<10^th^ percentile)	1239 (10.9)	616 (11.2)	623 (10.6)	0.32
Body mass index (BMI, kg/m^2^)	23.1±3.1	23.2±2.9	22.9±3.2	<0.0001
BMI grouping				0.72
<18.5 (underweight)	1224 (10.2)	599 (10.1)	625 (10.2)	
18.5–23.9 (normal weight)	6972 (57.8)	3434 (58.1)	3538 (57.5)	
24.0–27.9 (overweight)	3179 (26.4)	1559 (26.4)	1620 (26.4)	
≥28.0 (obese)	690 (5.7)	324 (5.5)	366 (6.0)	
Systolic blood pressure, mmHg	123.6±13.7	125.3±12.7	122.0±14.5	<0.0001
Diastolic blood pressure, mmHg	79.0±8.7	80.1±8.4	78.0±8.8	<0.0001
Hypertension	1693 (14.0)	824 (13.9)	869 (14.1)	0.75
Anti-hypertensive medication	477 (4.0)	198 (3.4)	279 (4.5)	0.001

Data presented are mean±SD for continuous variables, and n (%) for frequency variables.

* P values in t tests for differences in means or Chi-square tests for differences in proportions between men and women.

Compared to the non-exposed cohort adjusting for participant’s characteristics, SBP/DBP was about 0.7/0.7 mmHg higher in subjects exposed to famine during fetal development only overall (significantly for DBP, p<0.01; not significantly for SBP, p = 0.14), about 3.0/1.6 mmHg higher in those exposed to famine during infancy only (p<0.001), and about 1.5/0.6 mmHg higher in those exposed during both fetal development and infancy (p<0.001) (**Table 3**). The risk of hypertension was about 1.8-fold higher in subjects exposed to famine during infancy only (p<0.001), and 1.3-fold higher in subjects exposed during both fetal development and infancy (p<0.001), but was not significantly elevated in those exposed during fetal development only overall (p = 0.15). The adjusted effect estimates for famine exposure were similar with or without adjustment for BMI, and in normal weight, or overweight and obese subjects (data not shown).

**Table 3:** Blood pressure, height and BMI by exposure to the 1959–1961 Chinese Great Famine during fetal development and infancy (<2 years postnatal).

Exposure to the famine	Not exposed(n = 4872)	Fetal development only(n = 1156)	Infancy only(n = 3126)	Fetal development and infancy(n = 2911)
**Blood pressure**				
SBP*, mmHg	124.2±0.4	124.9±0.5	127.2±0.4τ	125.7±0.4τ
Difference*	reference	0.7 (−0.2, 1.6)	3.0 (2.4, 3.6)τ	1.5 (0.9, 2.2)τ
DBP*, mmHg	80.2±0.3	80.9±0.3	81.8±0.3	80.8±0.3
Difference*	reference	0.7 (0.2, 1.3)‡	1.6 (1.2, 2.0)τ	0.6 (0.2, 1.0)‡
Hypertension	539 (11.1)	144 (12.5)	605 (19.4)τ	405 (13.9)τ
Crude OR	reference	1.15 (0.94, 1.40)	1.91 (1.68, 2.17)τ	1.31 (1.13, 1.50)τ
Adjusted OR*	reference	1.16 (0.95, 1.42)	1.83 (1.61, 2.08)τ	1.31 (1.14, 1.51)τ
**BMI,** * **kg/m^2^, males**	23.3±0.09	23.3±0.14	23.5±0.10	23.2±0.10
Difference*	reference	−0.03 (−0.30, 0.25)	0.12 (−0.08, 0.31)	−0.18 (−0.38, 0.02)
Obesity (≥28 kg/m^2^)	140 (5.9)	34 (5.8)	89 (5.9)	61 (4.2)†
Crude OR	reference	0.99 (0.67, 1.45)	1.00 (0.76, 1.31)	0.70 (0.52, 0.95)†
Adjusted OR*	reference	1.00 (0.68, 1.47)	1.02 (0.77, 1.35)	0.72 (0.53, 0.98)†
**BMI,** * **kg/m^2^, females**	22.6±0.28	22.6±0.30	22.8±0.28	22.7±0.28
Difference*	reference	−0.02 (−0.23, 0.28)	0.17 (−0.03, 0.38)	0.12 (−0.09, 0.33)
Obesity (≥28 kg/m^2^)	133 (5.3)	32 (5.6)	107 (6.7)	94 (6.4)
Crude OR	reference	1.07 (0.72, 1.59)	1.26 (0.97, 1.64)	1.23 (0.94, 1.62)
Adjusted OR*	reference	1.08 (0.72, 1.61)	1.21 (0.93, 1.58)	1.24 (0.94, 1.63)
**Height** * **,** **cm, males**	168.0±0.2	167.5±0.3	166.8±0.2τ	167.2±0.2τ
Difference*	reference	−0.5 (−1.0, 0.05)	−1.1 (−1.5, −0.8)τ	−0.8 (−1.1, −0.4)τ
Short (<10^th^ percentile)	212 (9.0)	64 (11.0)	203 (13.4)	137 (9.5)
Crude OR	reference	1.27 (0.94, 1.71)	1.59 (1.29, 1.95)τ	1.07 (0.86, 1.35)
Adjusted OR*	reference	1.31 (0.97, 1.76)	1.57 (1.28, 1.93)τ	1.12 (0.89, 1.40)
**Height,** * **cm, females**	158.7±0.5	158.3±0.5	157.5±0.5τ	157.9±0.5τ
Difference*	reference	−0.4 (−0.9, 0.1)	−1.2 (−1.5, −0.9)τ	−0.8 (−1.2, −0.5)τ
Short (<10^th^ percentile)	208 (8.3)	47 (8.2)	215 (13.4)τ	153 (10.5)†
Crude OR	reference	1.00 (0.72, 1.39)	1.71 (1.39, 2.09)τ	1.28 (1.02, 1.59)†
Adjusted OR*	reference	1.05 (0.75, 1.46)	1.66 (1.35, 2.04)τ	1.27 (1.02, 1.59)†

Data presented are adjusted least square mean±standard error, or adjusted mean difference (95% CI) for continuous outcomes (blood pressure, BMI, height), and n (%) or odds ratio (95% CI) for dichotomous outcomes (hypertension, obesity, short stature).

* Adjusted for socio-demographic and lifestyle characteristics (see Table 2); for blood pressure and hypertension, further adjusted for short stature and BMI; subjects with anti-hypertensive treatment were included in the models for hypertension, but excluded in the models for SBP and DBP.

† p<0.05,

‡ p<0.01,

τ p<0.001 for comparisons to the non-famine reference cohort. All p values were<0.05 in tests for the overall differences in mean blood pressure or the risk of hypertension across the four study groups.

SBP = systolic blood pressure; DBP = diastolic blood pressure; BMI = Body mass index; OR = odds ratio; CI = confidence interval.

Height was about 1.1 cm lower in men and 1.2 cm lower in women among subjects exposed to famine during infancy only, and was about 0.8 cm lower in both sexes among those exposed during both fetal development and infancy (all p<0.001), and was marginally lower in those exposed during fetal development only overall (p = 0.08 for men, p = 0.12 for women) (**Table 3**). For both sexes, subjects exposed to famine during infancy only had an adjusted odds ratio for short stature of about 1.6 (p<0.01). Females exposed to famine during both fetal development and infancy had significantly higher odds of short stature (adjusted OR = 1.27, p = 0.03), while males exposed to famine during fetal development only had marginally higher odds for short stature (adjusted OR = 1.31, p = 0.08). There were no significant differences in BMI or odds of obesity comparing any famine exposure group to the non-exposed cohort, except for lower odds of obesity in men exposed during both fetal development and infancy (adjusted OR = 0.72, p = 0.03).

**Table 4** presents the effects of exposure to the Chinese Great Famine during fetal development only by trimester of exposure on blood pressure, height and BMI. Surprisingly, subjects exposed to the Chinese Great Famine during the 1^st^ trimester only had significantly higher SBP, DBP and risk of hypertension [adjusted OR = 1.36 (1.03, 1.79)], while there were no significant changes among subjects exposed during both the 1^st^ and 2^nd^ trimester only or all the three trimesters of pregnancy only without subsequent exposure during infancy. Subjects exposed to the famine during all the three trimesters of pregnancy only were more likely to be short in stature among males only [adjusted OR = 1.94 (1.22, 3.02)]. There were no other significant effects observed on BMI or stature.

**Table 4:** Effects of exposure to the Chinese Great Famine *during fetal development only* by trimester of exposure on blood pressure, height and BMI.

Exposure to the famine	Not exposed(n = 4872)	Fetal development 1^st^ trimester only(n = 486)	Fetal development 1^st^ and 2^nd^ trimester(n = 351)	Fetal development all three trimesters(n = 319)
**Blood pressure**				
SBP*, mmHg	124.2±0.4	125.6±0.8†	123.3±0.9	125.2±0.9
Difference*	reference	1.4 (0.2, 2.7)†	−0.9 (−2.3, 0.6)	1.1 (−0.4, 2.6)
DBP*, mmHg	80.2±0.3	81.8±0.5τ	80.5±0.6	80.5±0.6
Difference*	reference	1.5 (0.7, 2.3) τ	0.2 (−0.7, 1.1)	0.2 (−0.8, 1.1)
Hypertension	539 (11.1)	70 (14.4) †	37 (10.5)	37 (11.6)
Crude OR	reference	1.35 (1.03, 1.77)†	0.95 (0.67, 1.35)	1.06 (0.74, 1.50)
Adjusted OR*	reference	1.36 (1.03, 1.79)†	1.00 (0.70, 1.44)	1.03 (0.71, 1.49)
**BMI,** * **kg/m^2^, males**	23.3±0.09	23.3±0.21	23.02±0.26	23.52±0.26
Difference*	reference	0.03 (−0.38, 0.42)	−0.30 (−0.78, 0.17)	0.20 (−0.28, 0.70)
Obesity (≥28 kg/m^2^)	140 (5.9)	14 (5.7)	9 (5.2)	11 (6.8)
Crude OR	reference	0.96 (0.54, 1.69)	0.87 (0.44, 1.74)	1.16 (0.62, 2.19)
Adjusted OR*	reference	1.00 (0.65, 1.72)	0.87 (0.43, 1.75)	1.20 (0.63, 2.29)
**BMI,** * **kg/m^2^, females**	22.6±0.28	22.5±0.50	22.4±0.52	22.6±0.53
Difference*	reference	−0.04 (−0.47, 0.40)	−0.10 (−0.61, 0.40)	0.10 (−0.42, 0.63)
Obesity (≥28 kg/m^2^)	133 (5.3)	9 (3.8)	14 (5.7)	9 (5.7)
Crude OR	reference	0.70 (0.35, 1.39)	1.53 (0.86, 2.71)	1.08 (0.54, 2.17)
Adjusted OR*	reference	0.71 (0.34, 1.42)	1.59 (0.90, 2.84)	1.07 (0.53, 2.14)
**Height** * **,** **cm, males**	168.0±0.2	168.0±0.4	167.5±0.5	166.9±0.5†
Difference*	reference	−0.03 (−0.8, 0.7)	−0.5 (−1.4, 0.4)	−1.1 (−2.0, −0.1)†
Short (<10^th^ percentile)	212 (9.0)	20 (8.1)	20 (11.5)	24 (14.8) †
Crude OR	reference	0.90 (0.56, 1.45)	1.32 (0.81, 2.15)	1.77 (1.12, 2.79)†
Adjusted OR*	reference	0.91 (0.56, 1.47)	1.33 (0.81, 2.17)	1.94 (1.22, 3.09)‡
**Height,** * **cm, females**	158.7±0.5	158.5±0.8	158.4±0.8	158.5±0.8
Difference*	reference	−0.3 (−1.02, 0.35)	−0.5 (−1.3, 0.3)	−0.3 (−1.1, 0.5)
Short (<10^th^ percentile)	208 (8.3)	26 (10.9)	10 (5.7)	11 (7.0)
Crude OR	reference	1.35 (0.88, 2.07)	0.66 (0.34, 1.27)	0.83 (0.44, 1.56)
Adjusted OR*	reference	1.42 (0.92, 2.20)	0.69 (0.36, 1.33)	0.86 (0.46, 1.62)

Data presented are adjusted least square mean±standard error, or adjusted mean difference (95% CI) for continuous outcomes (blood pressure, BMI, height), and n (%) or odds ratio (95% CI) for dichotomous outcomes (hypertension, obesity, short stature).

* Adjusted for socio-demographic and lifestyle characteristics (see Table 2); for blood pressure and hypertension, further adjusted for short stature and BMI; subjects with anti-hypertensive treatment were included in the models for hypertension, but excluded in the models for SBP and DBP.

† p<0.05,

‡ p<0.01,

τ p<0.001 for comparisons to the non-famine reference cohort.

SBP = systolic blood pressure; DBP = diastolic blood pressure; BMI = Body mass index; OR = odds ratio; CI = confidence interval.

There were no significant differences in sex ratio of offspring among study groups (p = 0.55).

## Discussion

### Main Findings

An important new finding from the present study is that changes in exposure to famine during the fetal development period and from prenatal to early postnatal life may play a complex role in developmental programming the risk of hypertension in humans. The risk of hypertension was not elevated in subjects exposed to the Chinese Great Famine during fetal development only overall, but was significantly higher among those exposed during the first trimester of pregnancy only, and among those exposed during infancy only, as well as among those exposed during both fetal development and infancy. Improved infant nutrition following poor fetal nutrition appears to be beneficial, rather than harmful as suggested by the early postnatal nutrition “mismatch” theory [6].

### Comparisons with Previous Findings

The Dutch famine studies have reported inconsistent findings concerning the impact of exposure to famine during fetal development on the risk of hypertension in adulthood [7], [10], [11]. We speculated that this could be partly due to variable accuracy in clearly defining the exposure among studies, or that the relatively short duration of the Dutch Famine might have provided an opportunity for partially “recovering” in early postnatal life with better infant nutrition. This is supported by findings in animal models. For example, low nephron numbers may be a link between low birth weight and hypertension in adulthood [29], while a normal postnatal lactational environment restores nephron endowment and prevents later development of hypertension following placental restriction induced fetal growth restriction in rats [30]. It is unknown whether this finding may be applicable to humans. Epigenetic changes may be another mechanism in developmental programming, and critical epigenetic changes may occur well beyond fetal life [3]. As growth during the first two years of life remains relatively rapid and highly nutrition dependent, infancy may represent a critical window for further nutrition-induced epigenetic changes.

Two opposing theories exist concerning the role of infant nutrition in developmental programming of metabolic and cardiovascular risks. Large retrospective cohort (n>8000) data from Barker’s team strongly indicate that good nutrition during infancy (<2 years postnatal) is beneficial [21], [31], while data from two neonatal enriched nutrition trial cohorts of moderate size (n: 216 to 926) from Lucas’s team strongly suggest a deleterious effect of “mismatched” good infant nutrition following poor fetal nutrition [6], [20]. Data from our large southern Chinese quasi-experimental cohort support the beneficial impact of good nutrition during infancy. Exposure to famine during fetal development only overall or during the 1^st^ and 2^nd^ trimester only was not associated with higher risk of hypertension, but when exposure to famine during fetal development was followed by exposure to famine during infancy, the risk was about 30% higher. Average diastolic blood pressure was slightly higher in subjects exposed to famine during fetal development only overall. Risk analyses by trimester-specific exposure revealed that the increase in blood pressure was restricted to subjects exposed to famine during the first trimester of pregnancy only. There was a significantly increased risk of hypertension in these subjects exposed to famine during the first trimester of pregnancy only with no subsequent exposure in the remaining fetal development period. We speculate that exposure to famine during early gestation may be a critical period in “conditioning” the fetus to poor intrauterine environment, and subsequent “mismatched” better intrauterine environment may “program” the risk of hypertension. Surprisingly, those exposed during both the 1^st^ and 2^nd^ trimester only or all three trimesters of pregnancy only without subsequent exposure during infancy were no longer at increased risk of hypertension. One potential mechanism may be due to effect modifications by better postnatal nutrition, but why this does work for subjects exposed during the 1^st^ trimester of pregnancy only? We speculate that this could be due to a particularly deleterious “programming” effect on the risk of hypertension by a dramatic change from a poor to better (mismatched) nutritional environment from the 1^st^ to 2^nd^ trimester of pregnancy. Interestingly, exposure to famine during infancy only was associated with an even greater (about 80%) increased risk, suggesting that poor infant nutrition following “better” fetal nutrition is even worse. We speculate that this could be due to an unexpected “mismatch” shock in early postnatal life as infants who experienced good fetal nutrition had expected a more “friendly” postnatal environment. The observed effects of famine exposure in early life were independent of adult lifestyle factors or obesity.

There have been two recent Chinese studies on the effects of famine at younger ages on adult hypertension, but neither study estimated the effects of exposure to famine during fetal development only. Using data from 7874 participants in the 2002 China National Nutrition and Health Survey (mean age = 42), Li and colleagues observed that the relative risk of hypertension was 1.9 in subjects exposed to famine during fetal development, defined as those born from October 1959 through September 1961, as compared to control subjects (those born from October 1962 through September 1964) in areas of severe famine, but the risk was not elevated in areas of less severe famine (adjusted OR = 0.86, p>0.05) [17]. However, under our exposure classification system, most of the subjects which they considered to be exposed during fetal development would have been classified as exposed during both fetal development and infancy. By contrast, we observed a smaller but significantly higher (OR = 1.3) risk of adult hypertension in subjects exposed to famine during both fetal development and infancy, in a “less severe” famine region. In a study of 35,025 women using data from the 1993-1996 China-U.S. Collaborative Project for Neural Tube Defects Prevention (mean age = 32), Huang and colleagues reported that the risk of hypertension was three times as high among those born in 1958, but was not elevated among those born in 1957 or during the three famine years (1959–1961) as compared to subjects born in 1963 [18]. Under our exposure classification system, they would most likely have reported no effect in subjects exposed to famine during both fetal development and infancy, opposite to our findings and those of Li et al., but a larger effect in a subset of patients (the 1958 cohort) who were exposed to famine during infancy only – consistent with our observation that exposure to famine during infancy only might have a detrimental programming effect.

The Dutch famine studies have reported some sex- or gestational age-dependent effects of early life exposure to famine on BMI or obesity in adulthood: exposure during the first half of pregnancy increased the risk of obesity but exposure during the second half of pregnancy and early postnatal life reduced the risk of obesity [25], while exposure during early gestation might increase BMI in females only [26]. We did not observe any significant effects on BMI for exposure to famine during fetal development only overall, or during the first trimester only, or during both the first and second trimester of pregnancy only. A recent Chinese study reported that early postnatal exposure to famine increased adult BMI, but “fetal” (which actually included infant) exposure to famine decreased BMI [18]. Another recent Chinese study reported an increased risk of obesity in subjects exposed to famine during the first three years of life in females only [24]. By contrast, the risk of adult obesity was not elevated for any early life exposure to famine in our Southern Chinese birth cohort. There was even a modestly reduced risk of obesity in male subjects exposed to famine during both fetal development and infancy. The reasons behind the differences in these findings are unclear. Our data do not support the hypothesis that malnutrition in early life increases the risk of obesity in adulthood. To the contrary, it may decrease the risk of obesity, but still increase the risk of hypertension in adulthood.

A recent study reported shorter stature among subjects exposed to the Chinese Great Famine during the first three years of life for both sexes, but in females only among those exposed to famine during fetal development (but note that most subjects were also exposed to famine during infancy) [24]. Another recent study in women reported a decrease in adult height among those exposed to the Chinese Great Famine during the postnatal period only [18]. Our study confirmed the negative impact of famine during early postnatal life on stature. We further observed that exposure to famine during both fetal development and infancy had a stronger negative impact on stature in females, while exposure to famine during all the three trimesters of pregnancy only increased the risk of short stature in males only. The mechanisms are unclear. We speculate that some fetal growth regulation factors might be more vulnerable to nutritional disturbances in males or females during fetal and early postnatal life, dependent on the window of exposure.

### Strengths and Limitations

Our study is the first report on the risk of adult hypertension among subjects of about 50 years of age who were exposed to the Chinese Great Famine. Information collected from local residents allowed us to more precisely define the duration of famine in the study areas, thus for the first time to distinguish subjects who were exposed to the famine during fetal development only versus those who were exposed during both fetal development and infancy. Still, there may have been some misclassification of exposure since gestational age was unavailable for births in China during these years. However, such misclassifications would tend to attenuate the differences and bias the associations towards the null. It should be noted that all subjects exposed to famine during fetal development only were also exposed during the preconception period. However, we did not observe any increased risk of hypertension in subjects exposed during all the three trimesters of pregnancy plus the preconception period only without subsequent exposure during infancy. Similar to previous Chinese famine studies, we had no data on individual birth weight and gestational age. The effect estimates are relatively crude, approximating the typical effects for births in the famine years. Plausibly, there could have been more frequent low birth weight infants during the famine years. However, it can be argued that birth weight should not be adjusted for in estimating the health effects of famine because low birth weight or poor fetal growth could be considered a direct effect of famine on the causal pathway to the increased risk of hypertension. We had no data on family history of hypertension. However, there were unlikely significant changes in population genetic susceptibility among the study birth cohort subgroups spanning only several years.

### Conclusions

Our quasi-experimental study on the effects of early life exposure to the Chinese Great Famine strongly suggests a critical role for changes in exposure to famine during the fetal development period and from prenatal to postnatal life in developmental “programming” cardiovascular risk. Good infant nutrition appears to be beneficial *whatever* the fetus experienced in *utero*.
